# The complete mitochondrial genome of the Songpu mirror carp, *Cyprinus carpio*

**DOI:** 10.1080/23802359.2016.1143337

**Published:** 2016-02-10

**Authors:** Quan Huang, Zhen Wang

**Affiliations:** a College of fisheries, Huazhong Agricultural University, China;; b College of Animal Science and Technology, Jilin Agricultural University, Jilin, China

**Keywords:** Mitochondrial genome, phylogenetic analysis, Songpu mirror carp

## Abstract

In this study, the complete mitochondrial genome sequences of *Cyprinus carpio* (strain Songpu) were reported for the first time. The genome is found to be 16 582 bp in length accurately using the next-generation sequencing technology and bioinformatics tools, and it has a base composition of A (31.9%), G (15.7%), C (27.5%) and T (24.9%), indicating that the percentage of A + T (56.8%) was higher than G + C (43.2%). The whole genome contains a typically conserved structure, which includes 13 protein-coding genes, 22 transfer RNA genes, two ribosomal RNA genes and one control region (D-loop). To obtain the phylogenetic relationship between Songpu mirror carp and other species, 11 mitochondrial genomes were used for phylogenetic analysis. The result shows the Songpu mirror carp is closely related to *Cyprinu carpio carpio* that is also a member of the genus Cyprinu.

Carp (cyprinids) have been considered the most economically important teleost family and eco-friendly fish. *C. carpio*, as one of the dominant cyprinid species, is cultured in over 100 countries worldwide and produce over 3 million metric tons (Xu et al. 2014). In this study, we report the complete mitochondrial genome sequence of Songpu mirror carp (*C. carpio*) for the first time.

Genomic DNA was obtained from a homozygous double-haploid clonal line from the domesticated strain Songpu, and total genomic DNA was extracted and sequenced using the next-generation sequencing technology (Xu et al. 2014). We downloaded the genome raw reads, which were sequenced by Illumina HiSeq 2000 from NCBI (http:/www.ncbi.nlm.nih.gov/sra/). Sequenced data were then filtered and assembled into the complete mitochondrial genome with the CLC Genomic Workbench v3.6 (2010). The genome annotation was performed with Dual Organellar GenoMe Annotator (http://dogma.ccbb.utexas.edu). We chose default parameters to predict protein-coding genes (PCGs), transfer RNA genes (tRNA) and ribosomal RNA genes (rRNA) (Wyman et al. 2004). The complete genome sequence with all genes annotated was submitted to GenBank under the accession number of KU050703. To obtain the phylogenetic relationship between Songpu mirror carp and other species, 11 mitochondrial genomes were used for phylogenetic analysis. The complete mitochondrial genome sequences of 10 other species are available and downloaded from GenBank and the names and GenBank accession numbers of these species are as follows: *Campostoma anomalum* (NC_008102.1), *Biwia zezera* (NC_008324.1), *Catla catla* (NC_016892.1), *C. carpio xingguonensis* (NC_018036.1), *C. carpio haematopterus* (NC_018037.1), *C. carpio wuyuanensis* (NC_018039.1), *C. carpio carpio* (NC_018035.1), *B. springeri* (NC_022188.1), *Belligobio nummifer* (NC_023975.1), *C. carpio color* (NC_018366.1) (Wang et al. 2013).

The complete mitogenome sequence of Songpu mirror carp was 16 582 bp, consisting of 13 PCGs, 22 tRNA genes, two rRNA genes (12S rRNA gene and 16S rRNA), and one control region. All of the PCGs except nicotinamide adenine dinucleotide (NADH) dehydrogenase subunit 6 were encoded on the heavy strand. The whole genome base composition was estimated to be 31.9% A, 27.5% C, 15.7% G and 24.9% T, which is similar to many other teleost mitochondrial genomes with A/T bias (Wang et al. 2013). The putative control region is located between tRNA^Pro^ and tRNA^Phe^ with 927 bp in length.

Multiple alignments of 11 mitochondrial genomes sequence were performed using ClustalW with the default settings (Thompson et al. 1994). The phylogenetic tree from maximum-likelihood was reconstructed using the MEGA6 (Tamura et al. 2013). To get the confident supports, one thousand bootstrap replicates were set for each analysis. Phylogenetic analysis showed that 4 three clades within Figure 1. The Songpu mirror carp is closely related to *C. carpio carpio* that is also a member of the genus Cyprinu. The genus Cyprinu has a close relationship with the genus Catla.

**Figure 1.**
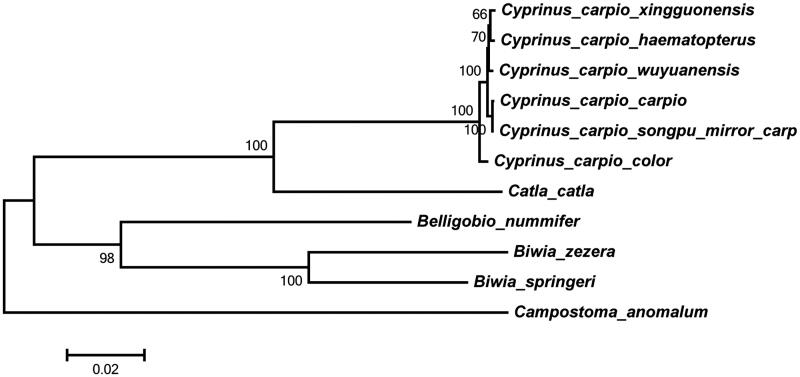
Phylogeny based on the complete mitochondrial genomes by MEGA6 with 1000 bootstrap replications. The maximum-likelihood tree is drawn to scale.
